# Transcatheter versus Isolated Surgical Aortic Valve Replacement in Young High-Risk Patients: A Propensity Score-Matched Analysis

**DOI:** 10.3390/jcm10153447

**Published:** 2021-08-03

**Authors:** Markus Mach, Thomas Poschner, Waseem Hasan, Tillmann Kerbel, Philipp Szalkiewicz, Ena Hasimbegovic, Martin Andreas, Christoph Gross, Andreas Strouhal, Georg Delle-Karth, Martin Grabenwöger, Christopher Adlbrecht, Andreas Schober

**Affiliations:** 1 Department of Cardiac Surgery, Medical University of Vienna, 1090 Vienna, Austria; thomas.poschner@meduniwien.ac.at (T.P.); tillmann.kerbel@meduniwien.ac.at (T.K.); n1405273@students.meduniwien.ac.at (P.S.); ena.hasimbegovic@meduniwien.ac.at (E.H.); martin.andreas@meduniwien.ac.at (M.A.); christoph.gross@meduniwien.ac.at (C.G.); 2 Department of Cardio-Vascular Surgery, Hospital Floridsdorf and Karl Landsteiner Institute for Cardio-Vascular Research, 1210 Vienna, Austria; martin.grabenwoeger@gesundheitsverbund.at; 3 Faculty of Medicine, Imperial College London, London SW7 2AZ, UK; waseem.hasan15@imperial.ac.uk; 4 Division of Cardiology, Department of Internal Medicine II, Medical University of Vienna, 1090 Vienna, Austria; 5 Center of Medical Physics and Biomedical Engineering, Medical University of Vienna, 1090 Vienna, Austria; 6 Department of Cardiology, Hospital Floridsdorf and the Karl Landsteiner Institute for Cardiovascular and Critical Care Research Vienna, 1210 Vienna, Austria; andreas.strouhal@gesundheitsverbund.at (A.S.); georg.delle-karth@gesundheitsverbund.at (G.D.-K.); c.adlbrecht@imed19.at (C.A.); andreas.schober@gesundheitsverbund.at (A.S.); 7 Faculty of Medicine, Sigmund Freud University, 1020 Vienna, Austria; 8 Imed19, Private Research Center, 1190 Vienna, Austria

**Keywords:** TAVI, TAVR, SAVR, aortic stenosis, young

## Abstract

Background: Younger patients with severe symptomatic aortic stenosis are a particularly challenging collective with regard to the choice of intervention. High-risk patients younger than 75 years of age are often eligible for both the transcatheter aortic valve replacement (TAVR) and the isolated surgical aortic valve replacement (iSAVR). Data on the outcomes of both interventions in this set of patients are scarce. Methods: One hundred and forty-four propensity score-matched patients aged 75 years or less who underwent TAVR or iSAVR at the Hietzing Heart Center in Vienna, Austria, were included in the study. The mean age was 68.9 years (TAVR 68.7 vs. SAVR 67.6 years; *p* = 0.190) and the average EuroSCORE II was 5.4% (TAVR 4.3 [3.2%] vs. iSAVR 6.4 (4.3%); *p* = 0.194). Results: Postprocedural adverse event data showed higher rates of newly acquired atrial fibrillation (6.9% vs. 19.4%; *p* = 0.049), prolonged ventilation (2.8% vs. 25.0%; *p* < 0.001) and multi-organ failure (0% vs. 6.9%) in the surgical cohort. The in-hospital and 30-day mortality was significantly higher for iSAVR (1.4% vs. 13.9%; *p* = 0.012; 12.5% vs. 2.8%; *p* = 0.009, respectively). The long-term survival (median follow-up 5.0 years (2.2–14.1 years)) of patients treated with the surgical approach was superior to that of patients undergoing TAVR (*p* < 0.001). Conclusion: Although the survival analysis revealed a higher in-hospital and 30-day survival rate for high-risk patients aged ≤75 years who underwent TAVR, iSAVR was associated with a significantly higher long-term survival rate.

## 1. Introduction

Treating non-geriatric patients with symptomatic aortic stenosis and a high surgical risk profile is challenging in light of the scarcity of data in this patient collective. This collective of patients has not yet been investigated in large randomized trials. Comparing the transcatheter aortic valve replacement (TAVR) to the isolated surgical aortic valve replacement (SAVR), the question of whether young high-risk patients who undergo TAVR share the excellent outcomes of recently published large clinical trials in the field remains unanswered [1,2].

Over the last decade, TAVR has evolved from an initially experimental procedure to a standard therapy option for severe aortic stenosis and is being performed more frequently than SAVR in some countries [2,3,4,5]. TAVR is currently indicated for patients suffering from severe symptomatic aortic valve stenosis who are at a high or intermediate surgical risk [6,7,8,9]. Furthermore, it has been suggested that TAVR is a safe and suitable option for patients at lower surgical risk levels [10,11]. As the list of possible indications is getting longer, the focus is shifting to concerns regarding prosthesis durability, periinterventional and postinterventional adverse outcomes and patient selection [12,13,14].

We investigated a cohort of patients under 75 years of age with significant comorbidities and a high surgical risk with symptomatic severe aortic stenosis who underwent either SAVR or TAVR. We performed propensity score matching and investigated the short- and long-term outcomes and procedural differences between TAVR and SAVR.

## 2. Materials and Methods

### 2.1. TAVR Cohort

This retrospective analysis was approved by the institutional Ethics Committee of the City of Vienna (EK 20-141—VK). Data from 532 patients enrolled in the Vienna Cardiothoracic Aortic Valve Registry (VICTORY) Registry at the Hietzing Heart Center from June 2009 to December 2016 were reviewed. One hundred and twenty-four patients aged 75 years or less were selected from this collective for further analysis. The 75-year cut-off was chosen according to the treatment allocation recommendations of the 2017 ESC/EACTS guidelines. Although no lower age limit was applied to the analysis, no patients younger than 53 years were included in the study. Patients who exceeded a EuroSCORE II of 4% or a logistic EuroSCORE of 10% were deemed to be at an increased risk for postoperative morbidity or mortality [6]. Each patient was assessed by the institutional Heart Team. The decision to treat these patients with TAVR was based on the risk factors and comorbidities listed in Table 1. Due to existing contraindications to SAVR, 16 patients were excluded from the analysis. Of the remaining 88 patients, 42 were treated via the percutaneous transfemoral and 46 via the transapical access site as previously described [15]. Different generations of transcatheter valves developed by Edwards Lifesciences (Edwards Lifesciences, Irvine, CA, USA), Medtronic (Medtronic, Minneapolis, MN, USA), JenaValve (JenaValve Technology GmbH, Munich, Germany) and Symetis (Symetis SA, a Boston Scientific company, Ecublens, Switzerland) were used.

### 2.2. iSAVR Cohort

Between January 2005 and December 2016, 732 patients younger than 75 years underwent iSAVR without concomitant procedures at the Department of Cardiovascular Surgery, Heart Center Hietzing (Vienna, Austria). iSAVR was performed according to standard surgical practice. A total of 128 patients were excluded from the analysis due to active endocarditis (*n* = 54) or incomplete datasets (*n* = 74). Thus, 604 iSAVR patients were deemed eligible for this study. Patients undergoing aortic valve replacement via a homograft implantation or Ross procedure were excluded from the analysis.

### 2.3. Study Design and Endpoint Definitions

**Figure 1:**
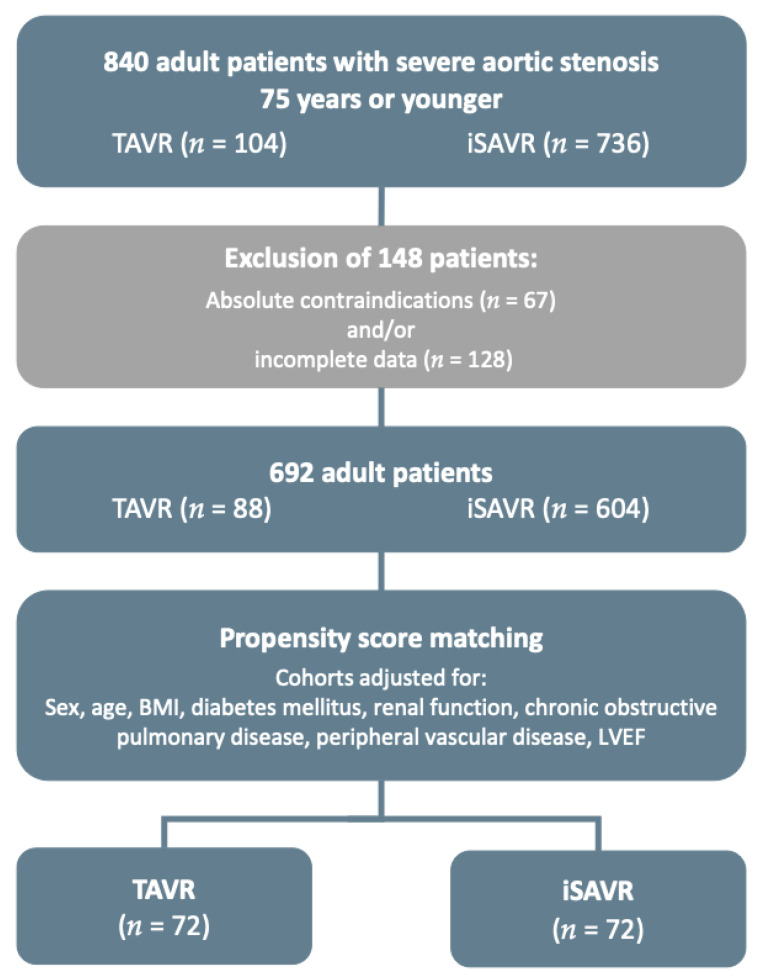
Patient selection and propensity score matching flow-chart (BMI—body mass index, LVEF—left ventricular ejection fraction).

A propensity score-matched analysis stratified for differences in the patients’ baseline characteristics was created to compare the outcome of patients undergoing iSAVR or TAVR. A flowchart depicting patient selection and statistical analysis is shown in Figure 1.

The primary study endpoints were defined as 30-day all-cause mortality and freedom from all-cause mortality after 5 years. The secondary endpoints were the occurrence of adverse events and peri- and postprocedural complications as set out by the updated Valve Academic Research Consortium (VARC)-II criteria including bleeding events, access-related vascular complications, myocardial infarction, acute kidney injury, neurological adverse events, the necessity of pacemaker implantation and reoperations [16].

### 2.4. Statistical Analysis 

Continuous variables were expressed as either the median and interquartile range (IQR) or as mean and standard deviation (±SD), based on their distribution. Categorical variables were expressed as absolute numbers and percentages and compared with the chi^2^ test or Fisher’s exact test.

### 2.5. Propensity Score Matching

**Figure 2:**
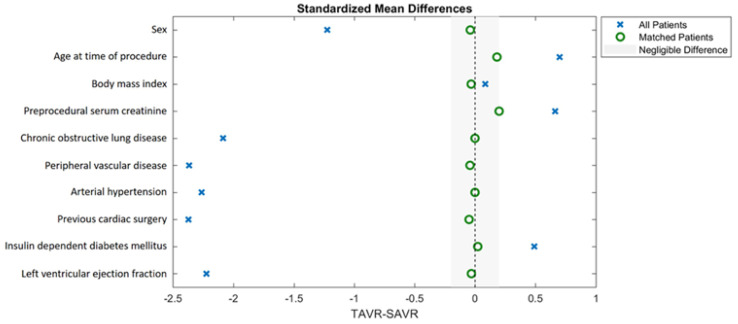
Standardized mean differences in matching variables before and after propensity score matching.

Propensity score matching was performed according to the recommendations proposed by McMurry et al. [17]. A non-parsimonious multivariable logistic regression model was used to calculate the propensity score. Rigorous adjustment for significant differences in the patients’ baseline characteristics relevant for the treatment assignment and potential outcomes was performed with 1-to-1 matching using the following algorithm: nearest neighbor matching with a caliper width of 0.1 standard deviation of the propensity score and no replacement. The propensity score model was adjusted for differences in the following baseline characteristics: sex, age at time of procedure, body mass index (BMI), preprocedural serum creatinine level, chronic obstructive lung disease, peripheral vascular disease, arterial hypertension, previous cardiac surgery, insulin-dependent diabetes mellitus and left ventricular ejection fraction. The average absolute standardized difference was 1.5 and 0.04 after matching (Figure 2).

Differences in categorical variables between the matched cohorts were analyzed with McNemar’s test, and continuous variables were compared using the Wilcoxon signed-rank test or paired samples t-test based on variable distribution. A Kaplan–Meier estimate of long-term survival was performed, and survival curves were compared by the test described by Klein and Moeschberger [18]. Baseline, procedural and outcome characteristics of patients excluded from the analysis after propensity score matching are summarized in Supplementary Tables S1–S3.

All reported *p*-values were two-sided, and the results were categorized as statistically significant with an alpha level set at <0.05; due to the exploratory nature of the analyses, *p*-values may be interpreted as descriptive rather than confirmatory. All analyses were performed using SPSS, version 24.0 (IBM Corp, Armonk, NY, USA).

## 3. Results

### 3.1. Baseline Characteristics

Baseline characteristics, procedural characteristics and adverse events for the unmatched and matched population are presented in Table 2, Table 3 and Table 4, respectively. Eighty-eight TAVR patients and 604 iSAVR patients were included in the retrospective analysis (64.1 ± 9.5 years, 287 (41.5%) female, median follow-up 5.5 years (2.2–14.1 years)). After propensity score matching, 72 matched pairs were compared. The cohorts did not differ regarding baseline characteristics used for adjustment in the analysis model (Figure 2). No differences were observed in the EuroSCORE II (4.3 ± 3.2% vs. 6.4 ± 4.3%; *p* = 0.194). However, patients undergoing TAVR had more often been treated with a percutaneous coronary intervention (PCI) prior to the procedure (TAVR: 19.4% vs. iSAVR: 5.6%; *p* = 0.021).

### 3.2. Survival and Safety Outcome 

After propensity score matching, a significant difference in ventilation times (TAVR: 4 ± 7 h vs. iSAVR: 12 ± 27 h; *p* < 0.001) was observed. Patients undergoing iSAVR demonstrated higher rates of new-onset atrial fibrillation (TAVR: 6.9% vs. iSAVR: 19.4%; *p* = 0.049), sepsis (TAVR: 0% vs. iSAVR: 2.8%, *p* = n/a) and pronounced wound infection (TAVR: 0% vs. iSAVR: 5.6%, *p* = n/a). Of the 43 patients (59.7%) receiving transapical TAVR only 6 patients (8.3%) were extubated in the operating theatre. Prolonged ventilation times of longer than 6 h were more frequent in the iSAVR cohort (TAVR: 2.8% vs. iSAVR: 25%; *p* < 0.001). Conduction disorders and pacemaker implantation only occurred in the TAVR cohort, and the overall incidence was exceptionally low (TAVR: 0% vs. iSAVR 2.8%, *p* = n/a). Major vascular access complications occurred only in patients treated with TAVR (TAVR: 5.6% vs. iSAVR 0.0%, *p* = n/a), resulting in a higher re-operation rate for non-cardiac causes (TAVR 6.9% vs. iSAVR 0%, *p* = n/a). Multi organ dysfunction syndrome, in-hospital death and 30-day all-cause mortality were significantly higher in the iSAVR cohort ([iSAVR: 0% vs. TAVR: 6.9%, *p* = n/a]; [1.4% vs. 13.9%; *p* = 0.012]; [2.8% vs. 12.5%; *p* = 0.022]).

**Figure 3:**
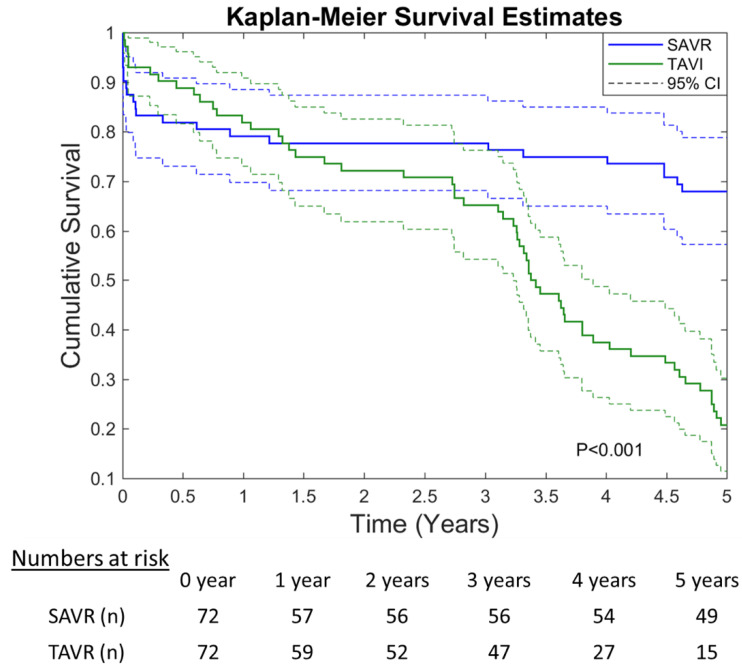
Five-year Kaplan–Meier survival curves (CI—Confidence Interval).

The 5-year Kaplan–Meier curve is depicted in Figure 3. Although TAVR was associated with an improved 30-day survival, iSAVR patients had higher long-term survival rates (*p* < 0.001).

## 4. Discussion

This study is the first propensity-matched comparison of TAVR and iSAVR in non-geriatric high-risk patients. Although the 30-day survival is higher after TAVR, iSAVR is linked to a higher long-term survival rate. This study confirms the known strengths and weaknesses of the respective therapy options: on the one hand, iSAVR was associated with a higher incidence of new-onset atrial fibrillation and prolonged ventilation times, and on the other hand, TAVR patients had a higher incidence of associated vascular access complications. This led to the conclusion that, while for non-geriatric patients who are at a high risk of suffering significant adverse events or dying during or immediately following surgery, prohibitive risk of morbidity or mortality can be treated effectively with TAVR, those likely to recover from surgery benefit from iSAVR in the long run.

The observed mortality differences in young high-risk patients provide new insights compared to isolated reports from either high-risk or non-geriatric populations. The PARTNER 1A trial investigated outcomes in TAVR and SAVR in a high-risk cohort and found no differences in the 30-day and 5-year mortality [19,20]. The intermediate risk PARTNER 2 trial similarly showed no differences in 30-day mortality between the procedures [21]. The difference between these reported survival rates between the interventions and the results observed in our patients suggest a different pattern in the younger subgroup of the high-risk population. A higher rate of recovery from adverse events as well as the lower prevalence of frailty among non-geriatric patients may account for the higher long-term survival rate after SAVR, whereas the higher 30-day survival rates following TAVR are likely the result of the minimally invasive nature of the procedure.

Potential TAVR-related survival benefits in low-risk septuagenarians and octogenarians (average age 79 years) were studied in the Nordic NOTION trial and the GARY registry. Both studies found no differences in the 1-year mortality between TAVR and SAVR. Additionally, the NOTION trial showed no significant difference in the mortality between TAVR and SAVR at five years [14,22,23]. On the other hand, the most recent real-world analysis of the GARY registry, published by Beyersdorf et al., in 2021, showed a difference in the 5-year outcomes. The study, conducted on a propensity score-matched collective chosen from a total of 18010 patients (1820 TAVR vs. 1820 iSAVR), showed a significantly reduced long-term survival (hazard ratio 1.51, 95% confidence interval 1.35–1.68; *p* < 0.0001) after an implantation of early generation TAVI valves compared with SAVR [24].

Device improvements made between subsequent valve generations mainly aimed to reduce the paravalvular leakage rate by redesigning perivalvular skirts and reducing the size of delivery systems, and to improve access to the coronary ostia by increasing the cell size in the stent frame. Therefore, in light of the minimal changes made to the method of leaflet suspension and the anticalcification methods, and the consequently similar expected rates of structural valve deterioration, it is likely that similar survival curves might be seen after analysis of data with most recent valve designs. However, the PVL rate after TAVR has decreased significantly over the years and can have a substantial impact on long-term survival [25]. Among younger low-risk TAVR patients, whose mean age was closer to 74, the PARTNER 3 trial reported significantly lower rates of 30-day mortality or stroke for TAVR compared to SAVR, and the EVOLUT trial found that TAVR was noninferior with regard to death or disabling stroke at 24 months [10,11]. While our analysis seems to indicate a short-term survival benefit of TAVI in young high-risk patients, iSAVR is linked to a higher long-term survival rate, in contrast to the findings of the abovementioned trials. Although high-risk patients appear to share a few similarities with previously investigated populations, they require an individually tailored approach.

The differences in procedural outcomes between TAVR and iSAVR found in our analysis corroborate results from previous studies. iSAVR patients experienced significantly longer ventilating times and more instances where the ventilation time exceeded six hours. Prolonged ventilation times have been linked to delirium after cardiac surgery, which in turn has been shown to reduce the 30-day survival rate for both TAVR and SAVR [26,27]. New onset atrial fibrillation has consistently been found to occur more commonly after SAVR, which is in line with our findings [10,11,19,21]. Our TAVR cohort had a higher incidence of AV block and correspondingly higher rates of pacemaker implantation, both of which are in accordance with previous findings [10,22,28].

Certain differences in procedural outcomes between our cohorts are due to inherent differences between TAVR and iSAVR. Pronounced wound infections only occurred in the iSAVR cohort. The higher rates of major vascular access complications in the TAVR population are a consequence of the sheath size and manipulation during TAVR [19,21]. A higher incidence of major vascular access complications was not found in the NOTION, PARTNER 3 or EVOLUT trials [10,11,22]. The difference likely stems from the use of early-generation TAVR devices—the newer-generation valves used in these trials have low-profile sheaths.

This study has several limitations, most of them inherent to retrospective analysis. The single-center study with limited patient numbers may impede generalization of events yet emphasizes the necessity of larger randomized trials in this unusual, young high-risk population. The possibility of the presence of unidentified confounding variables cannot be excluded as the patients were not randomized. Propensity score matching cannot replace a prospective randomized analysis. The higher rate of certain comorbidities and potential other unidentified confounders including frailty may have played a role in the divergence of the survival curves after 1.5 years. Furthermore, individual patient preferences and the interdisciplinary decision-making process in the Heart Team may result in individual deviations from guideline recommendations.

Discrepancies between the results of our study and other trials may result from propensity score matching. For example, the 30-day mortality in our matched iSAVR cohort deviates from previously reported values (VICTORY: 12.5%, PARTNER 1A: 6.5%, PARTNER 2: 4.1%, PARTNER 3: 1.1%, EVOLUT: 1.3%, NOTION: 3.7%, GARY: 2.9%, Schaefer et al.: 1.1%) [10,11,14,19,21,26]. However, examining non-geriatric high-risk TAVR patients using propensity score matching inevitably resulted in an iSAVR cohort with an unusually high mortality rate compared to our overall iSAVR population and previous studies. The EuroSCORE II of the iSAVR population increased almost fourfold after propensity score matching (unmatched iSAVR: 1.7 vs. propensity score-matched iSAVR: 6.4) and the 30-day mortality increased almost five times (unmatched iSAVR 2.7% vs. propensity score-matched iSAVR 12.5%). Our results are more susceptible to biases caused by propensity score matching due to the small cohort size. However, the propensity score matching may have eliminated TAVR patients with numerous comorbidities and extremely high risk levels, as evidenced by the reduced EuroSCORE II (unmatched TAVR: 5.9 vs. matched TAVR: 4.3) and mortality (unmatched TAVR 3.4% vs. matched TAVR: 2.8%).

The use of risk scores to compare TAVR and iSAVR patients is problematic. TAVR patients often have substantially different risk profiles compared to iSAVR patients. Furthermore, not all relevant parameters are represented in existing surgical risk scores. As a result, despite analyzing a high-risk TAVR cohort, its EuroSCORE II was lower than that of the iSAVR cohort. Although frailty typically appears to be a minor contributing factor to postprocedural outcome in younger low-risk patients, the patients compared in our analysis were often significantly advanced in their biological age as a result of the higher number of comorbidities and the consecutively increased risk profile. Therefore, effectively comparing iSAVR and TAVR patients requires an appropriate scoring system that includes both the well-established traditional risk factors as well as frailty assessment tools.

## 5. Conclusions

Among younger high-risk patients, 30-day mortality rates are lower after TAVR, but the long-term survival is decidedly higher iSAVR. Other postprocedural outcomes were similar to patterns observed in other TAVR/SAVR studies. The only notable differences most likely stem from the use of first-generation TAVR devices and propensity score matching. Further research is required to determine when interventional procedures are futile and patients too frail for either procedure. Decisions regarding the method of aortic valve replacement should be led by the likelihood of surviving surgery and the immediate postoperative period in order for the patient to reap the long-term benefits of a surgical valve replacement.

## Figures and Tables

**Table 1:** Factors impacting the choice of TAVR over iSAVR.

TAVR < 75 Years *n* = 104	
Prohibitive surgical risk, n (%) ^1^	8 (7.7)
Porcelain aorta, n (%) ^1^	9 (8.7)
High-risk reoperation, n (%)	42 (40.4)
Respiratory impairment, n (%)	41 (39.4)
Severely reduced LVEF, n (%)	34 (32.7)
Severe renal insufficiency, n (%)	32 (30.8)
Substance abuse, n (%)	23 (22.1)
Adipositas per magna, n (%)	16 (15.4)
Valve-in-Valve procedure, n (%)	13 (12.5)
Neurological impairment, n (%)	12 (11.5)
Hepatopathy, n (%)	10 (9.6)
History of radiation to the chest, n (%)	9 (8.7)
Severe mental disorder, n (%)	9 (8.7)
Pulmonary hypertension, n (%)	7 (6.7)
Frailty, n (%)	3 (2.9)
Severe rhythm disorder, n (%)	2 (1.9)
History of severe bleeding, n (%)	1 (1.0)
Other, n (%)	17 (16.3)
Patients with 2 or more reasons listed above	74 (71.2)
Patients with 3 or more reasons listed above	35 (33.7)

^1^ Excluded from analysis due to absolute SAVR contraindications; LVEF—left ventricular ejection fraction; iSAVR—isolated surgical aortic valve replacement; TAVR—transcatheter aortic valve replacement.

**Table 2:** Baseline clinical characteristics of the unmatched and matched cohorts.

		Unmatched-Population (*n* = 692)		PS-Matched-Population (*n* = 144)		
	Overall *n* = 692	iSAVR < 75 Years *n* = 604	TAVR < 75 Years *n* = 88	iSAVR < 75 Years *n* = 72	TAVR < 75 Years *n* = 72	*p*-Value
Demographics						
Age, mean (±SD)	64.1 (9.5)	63.4 (9.8)	68.9 (5.2)	67.6 (7)	68.7 (5.5)	0.190
Female, n (%)	287 (41.5)	237 (39.2)	50 (56.8)	33 (45.8)	39 (54.2)	0.418
Body mass index kg/m^2^, median (IQR)	28.7 (5.5)	28.6 (5.4)	29.2 (6.5)	29.3 (4.8)	29.1 (6.7)	0.854
Risk profile						
EuroSCORE II, median (IQR)	2.7 (3.7)	1.7 (2.2)	5.9 (5.3)	6.4 (4.3)	4.3 (3.2)	0.194
Chronic Health Conditions and Risk Factors						
Hypertension, n (%)	538 (77.7)	464 (92.2)	74 (84.1)	53 (73.6)	62 (86.1)	0.089
Dyslipidaemia, n (%)	433 (62.6)	380 (82.3)	53 (60.2)	42 (36.2)	41 (35.3)	0.999
Diabetes mellitus, n (%)	200 (28.9)	164 (27.2)	36 (40.9)	9 (12.5)	8 (11.1)	0.371
Active smoker, n (%)	126 (18.2)	106 (17.5)	20 (22.7)	9 (12.5)	18 (25.0)	0.121
Serum creatinine mg/dL, mean (±SD)	1.1 (0.6)	1.0 (0.4)	1.5 (1.2)	1.4 (0.7)	1.3 (0.6)	0.894
Preoperative dialysis, n (%)	6 (0.9)	2 (0.3)	4 (4.5)	2 (2.8)	1 (1.4)	0.999
Chronic obstructive pulmonary disease, n (%)	217 (31.4)	168 (24.3)	49 (7.1)	41 (56.9)	37 (51.4)	0.608
Peripheral vascular disease, n (%)	67 (9.7)	43 (6.2)	24 (27.3)	14 (19.4)	14 (19.4)	0.999
Cerebrovascular disease, n (%)	111 (16.0)	87 (14.4)	24 (3.5)	9 (12.5)	19 (26.4)	0.031
Previous cerebrovascular accident, n (%)	17 (2.5)	7 (1.2)	10 (11.4)	4 (5.6)	9 (12.5)	0.227
Atrial fibrillation, n (%)	119 (17.2)	101 (16.7)	18 (20.5)	14 (19.4)	13 (18.1)	0.999
Previous myocardial infarction, n (%)	54 (7.8)	37 (6.1)	17 (19.3)	10 (13.9)	13 (18.1)	0.629
New York Heart Association class III/IV, n (%)	367 (53.1)	288 (47.7)	79 (90)	51 (70.8)	63 (87.5)	0.072
Preprocedural PCI, n (%)	43 (6.2)	26 (4.3)	17 (19.3)	4 (5.6)	14 (19.4)	0.021
Previous pacemaker implantation, n (%)	32 (4.6)	17 (2.8)	15 (17)	5 (6.9)	11 (15.3)	0.210
Previous cardiac surgery, n (%)	64 (9.2)	26 (4.3)	38 (43.2)	16 (22.2)	26 (36.1)	0.064
Previous CABG, n (%)	34 (4.9)	11 (1.8)	23 (26.1)	9 (12.5)	17 (23.6)	
Previous valve surgery, n (%)	34 (4.9)	16 (2.6)	18 (20.5)	10 (13.9)	10 (13.9)	
aortic, n (%)	25 (3.6)	12 (2.0)	13 (14.8)	9 (12.5)	10 (13.9)	
mitral, n (%)	9 (1.5)	4 (0.7)	5 (5.7)	1 (1.4)	0 (0)	
tricuspid, n (%)	3 (0.4)	2 (0.3)	1 (1.0)	0 (0)	0 (0)	
Previous other cardiac surgery, n (%)	17 (2.5)	2 (0.3)	15 (17)	0 (0)	10 (13.9)	
Preoperative Echocardiographic Data						
Mean pressure gradient, mean (±SD)	48 (17.3)	48.6 (17.6)	46.3 (18.3)	48.3 (17.9)	46.7 (18.6)	0.266
Left ventricular ejection fraction %, mean (±IQR) ^1^	52.7 (9.9)	53.4 (9.2)	46.5 (11.9)	51.7 (12.8)	47.7 (11.3)	0.061

^1^ McNemar (for binary variables) and Wilcoxon signed-rank test or paired samples *t*-test (for continuous variables); PS—propensity score; CABG—coronary artery bypass graft; EuroSCORE—European System for Cardiac Operative Risk Evaluation; IQR—interquartile range; PCI—percutaneous coronary intervention; PS—propensity score SD—standard deviation.

**Table 3:** Procedural characteristics of the unmatched and matched cohorts.

		Unmatched-Population (*n* = 692)		PS-Matched-Population (*n* = 144)		
	Overall *n* = 692	iSAVR < 75 Years *n* = 604	TAVR < 75 Years *n* = 88	iSAVR < 75 Years *n* = 72	TAVR < 75 Years *n* = 72	*p*-Value
Procedural Characteristics						
Biological valve prosthesis, n (%)	595 (86.0)	507 (83.9)	88 (100)	62 (86.1)	72 (100)	n/a ^‡^
Balloon-expandable THV, n (%)			56 (63.6)		43 (59.7)	n/a ^‡^
Prosthesis size in mm, mean (±SD)	23.2 (3.2)	22.8 (3.1)	26.3 (2.2)	22.7 (2.2)	26.5 (2.1)	<0.001
Full sternotomy, n (%)		494 (81.2)		66 (91.7)		n/a ^‡^
Cross-clamp time, mean (±SD)	58.3 (31)	58.3 (31)	0 (0)	62.8 (21.5)	0 (0)	n/a ^‡^
Perfusion time, mean (±SD)	87.8 (52.1)	87.8 (52.1)	0 (0)	111.2 (40.2)	0 (0)	n/a ^‡^
Transfemoral access, n (%)			42 (47.7)		29 (40.3)	n/a ^‡^
Predilatation, n (%)			43 (48.9)		38 (52.8)	n/a ^‡^
Postdilatation, n (%)			9 (10.2)		5 (6.9)	n/a ^‡^
Paravalvular leak > mild, n (%)	1 (0.1)	0 (0)	1 (1.1)	0 (0)	1 (1.4)	0.999
Postoperative circulatory support, n (%)	8 (1.2)	7 (1.2)	1 (1.1)	4 (5.6)	0 (0)	n/a ^‡^
Extubated in the operating room, n (%)	10 (1.4)	0 (0)	10 (12)	0 (0)	6 (8.3)	n/a ^‡^
Total hours ventilated, median (±IQR)	8 (8)	8.0 (8)	4 (7)	12 (27)	4 (7)	<0.001
Re-intubated during hospital stay, n (%)	22 (3.2)	19 (3.1)	3 (3.6)	4 (5.6)	3 (4.5)	0.999
Number of administered red blood cell units, mean (±SD)	0.6 (1.6)	0.6 (1.6)	0.6 (1.2)	1.0 (3.0)	0.6 (1.2)	0.242
Length of stay, median (±IQR)	11.0 (5)	11 (5)	9 (7)	11.5 (6)	9.0 (7)	0.188

n/a ^‡^—not calculated if a variable is constant in one cohort; PS—propensity score; IQR—Interquartile range; SD—standard deviation; THV—transcatheter heart valve.

**Table 4:** Adverse events in the unmatched and matched cohorts.

		Unmatched-Population (*n* = 692)		PS-Matched-Population (*n* = 144)		
	Overall *n* = 692	iSAVR < 75 Years *n* = 604	TAVR < 75 Years *n* = 88	iSAVR < 75 Years *n* = 72	TAVR < 75 Years *n* = 72	*p*-Value
VARC-2 Adverse Events						
Myocardial infarction, n (%)	2 (0.3)	1 (0.2)	1 (1.1)	0 (0)	1 (1.4)	n/a ^‡^
Neurological adverse event, n (%)	9 (1.3)	7 (1.2)	2 (2.3)	2 (2.8)	2 (2.8)	0.999
Major vascular access complication, n (%)	5 (0.7)	0 (0)	5 (5.7)	0 (0)	4 (5.6)	n/a ^‡^
Major bleeding complication, n (%)	28 (4.0)	24 (4.0)	4 (4.6)	6 (8.3)	3 (4.2)	0.508
Postoperative dialysis, n (%)	10 (1.4)	8 (1.3)	2 (2.3)	2 (2.8)	2 (2.8)	0.999
New-onset atrial fibrillation, n (%)	84 (12.1)	79 (13.1)	5 (5.7)	14 (19.4)	5 (6.9)	0.049
AV-Block III, n (%)	16 (2.3)	11 (1.8)	5 (5.7)	0 (0)	2 (2.8)	n/a ^‡^
Pacemaker implantation, n (%)	18 (2.6)	13 (2.2)	5 (5.7)	0 (0)	2 (2.8)	n/a ^‡^
Reoperation for valvular dysfunction, n (%)	2 (0.3)	2 (0.3)	0 (0)	1 (1.4)	0 (0)	n/a ^‡^
Reoperation for bleeding/tamponade, n (%)	16 (2.3)	15 (2.5)	1 (1.1)	4 (5.6)	1 (1.4)	0.375
Reoperation for other cardiac problem, n (%)	5 (0.7)	2 (0.3)	3 (0.4)	1 (1.4)	2 (2.8)	0.999
Reoperation for non-cardiac problem, n (%)	15 (2.2)	10 (1.7)	5 (5.7)	0 (0)	5 (6.9)	n/a ^‡^
Postoperative sepsis, n (%)	4 (0.6)	4 (0.7)	0 (0)	2 (2.8)	0 (0)	n/a ^‡^
Pronounced wound infection, n (%)	9 (1.3)	9 (1.5)	0 (0)	5 (5.6)	0 (0)	n/a ^‡^
Prolonged ventilation > 6 h, n (%)	64 (9.2)	61 (10.1)	3 (0.4)	18 (25.0)	2 (2.8)	<0.001
Multi- organ dysfunction syndrome, n (%)	10 (1.4)	10 (1.7)	0 (0)	5 (6.9)	0 (0)	n/a ^‡^
In-hospital death, n (%)	17 (2.5)	16 (2.3)	1 (1.1)	10 (13.9)	1 (1.4)	0.012
30-day all-cause mortality, n (%)	19 (2.7)	16 (2.7)	3 (3.4)	9 (12.5)	2 (2.8)	0.022

n/a ^‡^—not calculated if the variable is constant in one cohort; AV—atrioventricular.

## Data Availability

The datasets for this study will be made available from the corresponding author upon reasonable request.

## Supplementary Materials

The following are available online at https://www.mdpi.com/article/10.3390/jcm10153447/s1, Supplementary Table S1: Baseline clinical characteristics of the excluded cohorts; Supplementary Table S2: Procedural characteristics of the excluded cohorts; Supplementary Table S3: Adverse events of the excluded cohorts.
