# Supplementary material for: Transcatheter versus Isolated Surgical Aortic Valve Replacement in Young High-Risk Patients: A Propensity Score-Matched Analysis
Source: J Clin Med. 2021 Aug 3;10(15):3447. doi: 10.3390/jcm10153447 (PMC8346998; doi:10.3390/jcm10153447)
Supplement: Supplementary file 1 [file jcm-10-03447-s001.zip › jcm-1293659-supplementary.pdf]

Supplementary Table S1. Baseline clinical characteristics of the excluded cohorts

	Excluded after PSM n = 548		iSAVR < 75 years n = 532		TAVR < 75 years n = 16	
**Demographics**						
Age, mean-(±SD)	68.8	(5.6)	62.8	(10.0)	69.6	(4.2)
Female, n-(%)	215	(39.2)	204	(38.3)	11	(68.8)
Body mass index kg/m2, median-(IQR)	28.8	(5.8)	28.6	(5.5)	29.5	(5.3)
**Risk profile**						
EuroSCORE II, median (IQR)	4.6	(3.5)	1.5	(1.8)	13.4	(6.7)
**Chronic health conditions and risk factors**						
Hypertension, n (%)	423	(77.2)	411	(77.3)	12	(75.0)
Dyslipidaemia, n (%)	350	(63.9)	338	(63.5)	12	(75.0)
Diabetes mellitus, n (%)	43	(7.8)	39	(7.3)	4	(25.0)
Active smoker, n (%)	171	(31.2)	165	(31.0)	6	(37.5)
Serum creatinine mg/dL, mean (±SD)	1.3	(0.8)	1.0	(0.3)	2.4	(2.2)
Preoperative dialysis, n (%)	3	(0.5)	0	(0)	3	(18.8)
Chronic obstructive pulmonary disease, n (%)	139	(25.3)	127	(22.8)	12	(75.0)
Peripheral vascular disease, n (%)	39	(7.1)	29	(5.5)	10	(62.5)
Cerebrovascular disease, n (%)	83	(15.2)	78	(14.7)	5	(31.3)
Previous cerebrovascular accident, n (%)	4	(0.7)	3	(0.6)	1	(6.3)
Atrial fibrillation, n (%)	92	(16.8)	87	(16.4)	5	(31.3)
Previous myocardial infarction, n (%)	31	(5.7)	27	(5.1)	4	(25.0)
New York Heart Association class III/IV, n (%)	253	(46.2)	237	(44.6)	16	(100)
Previous PCI, n (%)	25	(4.7)	22	(4.1)	3	(18.8)

Previous pacemaker implantation, n (%)	16	(2.9)	12	(2.3)	4	(25.0)
Previous cardiac surgery, n (%)	22	(4.0)	10	(1.9)	12	(75.0)
Previous CABG, n (%)	8	(1.6)	2	(0.4)	6	(37.5)
Previous valve surgery, n (%)	14	(2.6)	6	(1.3)	8	(50.0)
Previous other cardiac surgery, n (%)	7	(58.3)	31	(0.4)	5	(31.3)
**Preoperative echocardiographic data**						
Mean pressure gradient, mean (±SD)	47.8	(19.0)	49.2	(24.3)	43.7	(17.1)
Left ventricular ejection fraction %, mean (±IQR)	45.8	(12.1)	53.8	(8.6)	41.1	(13.1)

CABG – Coronary Artery Bypass Graft; COPD – chronic obstructive pulmonary disease; EuroSCORE - European System for Cardiac Operative Risk Evaluation; IDDM – Insulin dependent diabetes mellitus; IQR – Interquartile range; LVEF - left ventricular ejection fraction; NYHA – New York Heart Association; PSM – propensity score matching; PCI - percutaneous coronary intervention; SD – standard deviation;

Supplementary Table S2. Procedural characteristics of the excluded cohorts

	Excluded after PSM n = 548		iSAVR < 75 years n = 532		TAVR < 75 years n = 16	
**Procedural variables**						
Biological valve prosthesis, n (%)	461	(84.1)	445	(83.6)	16	(100)
Balloon-expandable THV, n (%)					10	(62.5)
Prosthesis size in mm, mean (±SD)	26.0	(2.5)	22.8	(3.2)	25.4	(2.6)
Full sternotomy, n (%)			428	(80.5)		
Cross-clamp time, mean (±SD)			67.3	(23.4)		
Perfusion time, mean (±SD)			99.2	(42.9)		
Transfemoral access, n (%)					13	(81.3)
Predilatation, n (%)					5	(31.3)
Postdilatation, n (%)					4	(25)
Paravalvular leak > mild, n (%)	0	(0)	0	(0)	0	(0)
Postoperative circulatory support, n (%)	4	(0.7)	3	(0.6)	1	(6.3)
Extubated in the OR, n (%)	4	(0.7)	0	(0)	4	(25)
Total hours ventilated, median (±IQR)	5	(9)	8	(8)	4	(8)
Re-intubated during hospital stay, n (%)	15	(2.7)	15	(2.8)	0	(0)
Number of red blood cell units, mean (±SD)	0.9	(2.6)	1.0	(3.0)	0	(0)
Length of stay, median (±IQR)	10	(8)	11	(5)	10	(13)

OR - operating room; THV - transcatheter heart valves; other abbreviations as in Supplementary table 1

Supplementary Table S3. Adverse events of the excluded cohorts

	Excluded after PSM n = 548		iSAVR < 75 years n = 532		TAVR < 75 years n = 16	
**Procedural variables**						
Myocardial infarction, n (%)	1	(0.2)	1	(0.2)	0	(0)
Neurological adverse event, n (%)	4	(0.7)	4	(0.8)	0	(0)
Major vascular complication, n (%)	1	(0.2)	0	(0)	1	(6.3)
Major bleeding complication, n (%)	19	(3.5)	18	(3.4)	1	(6.3)
Postoperative renal replacement therapy, n (%)	6	(1.1)	6	(1.1)	0	(0)
New-onset atrial fibrillation, n (%)	65	(11.9)	65	(12.2)	0	(0)
AV-Block III, n (%)	16	(2.9)	13	(2.4)	3	(18.8)
Pacemaker implantation, n (%)	16	(2.9)	13	(2.4)	3	(18.8)
Reoperation for valvular dysfunction, n (%)	1	(0.2)	1	(0.2)	0	(0)
Reoperation for bleeding/tamponade, n (%)	11	(2.0)	11	(2.1)	0	(0)
Reoperation for other cardiac problem, n (%)	2	(0.4)	1	(0.2)	1	(6.3)
Reoperation for non-cardiac problem, n (%)	10	(1.8)	10	(1.9)	0	(0)
Postoperative sepsis, n (%)	2	(0.4)	2	(0.4)	0	(0)
Deep sternal wound infection, n (%)	5	(0.9)	5	(0.9)	0	(0)
Prolonged ventilation >6hrs, n (%)	44	(8.0)	43	(8.1)	1	(6.3)
Multi-system organ failure, n (%)	5	(0.9)	5	(0.9)	0	(0)
In-hospital death, n (%)	6	(1.1)	6	(1.3)	0	(0)
30-day all-cause mortality, n (%)	8	(1.6)	7	(1.3)	1	(6.3)

AV – atrioventricular; other abbreviations as in Supplementary table 1, 2 and 3
